# Sacubitril-Valsartan Increases Ultrafiltration in Patients Undergoing Peritoneal Dialysis: A Short-Term Retrospective Self-Controlled Study

**DOI:** 10.3389/fmed.2022.831541

**Published:** 2022-06-03

**Authors:** Fen Zhang, Tingting Zhang, Sisi Yang, Di Wang, Qianqian Zhuo, Xianhui Qin, Nirong Gong, Jun Ai

**Affiliations:** State Key Laboratory of Organ Failure Research, Guangdong Provincial Key Laboratory of Renal Failure Research, Guangzhou Regenerative Medicine and Health Guangdong Laboratory, National Clinical Research Center of Kidney Disease, Division of Nephrology, Nanfang Hospital, Southern Medical University, Guangzhou, China

**Keywords:** sacubitril-valsartan, ultrafiltration, blood pressure, peritoneal dialysis, short-term

## Abstract

**Aim:**

There are few data about the effectiveness and safety of angiotensin receptor-neprilysin inhibitor (ARNI) sacubitril-valsartan in end-stage renal disease (ESRD) patients undergoing peritoneal dialysis (PD). The present study was conducted to evaluate the association between sacubitril-valsartan treatment and peritoneal ultrafiltration (PUF) in PD patients.

**Methods and Results:**

Forty-seven ESRD patients undergoing PD for at least 3 months without severe congestive heart failure (CHF) were included in this study. Sacubitril-valsartan (generally 100 mg b.i.d) was administered after consultation with the nephrologist. Angiotensin-converting enzyme (ACE) inhibitors and angiotensin receptor blockers (ARBs) were required to be discontinued 36 h before prescribing sacubitril-valsartan. Other treatments and dialysis modality did not change. Baseline demographic and clinical parameters were collected before ARNI administration, and daily PUF, urine volume, total output, blood pressure (BP), and body weight were collected within 7 days before and after ARNI treatment. After treated with sacubitril-valsartan, 30 patients (63.8%) had a significant increase of PUF [up to 150.4 (110.7, 232.1) ml per day], while the remaining 17 (36.2%) had a slight decrease. The overall increase of PUF was 66.4 (21.4, 123.2) ml/24 h within the 7 days after sacubitril-valsartan administration, which was significantly higher than those before (*P* = 0.004). Total output, BP, and body weight also significantly improved. No adverse drug reactions were observed.

**Conclusions:**

Our study indicated that sacubitril-valsartan was associated with the increase of short-term PUF and total output in PD patients.

## Introduction

Sacubitril-valsartan, a first-inclass angiotensin receptor-neprilysin inhibitor (ARNI), is a sodium salt complex of sacubitril [a neprilysin inhibitor (NEPI)] and valsartan [an angiotensin receptor blocker (ARB)] in a 1:1 molar ratio (1–3). This complex may present significant advancement over angiotensin-converting enzyme (ACE) inhibition or ARB alone, because NEP inhibition acts synergistically with renin-angiotensin-aldosterone system (RAAS) blockade, which could dilate blood vessels, strengthen diuresis and natriuresis, prevent cardiac remodeling, and support cardiomyocyte survival (4, 5). In PARADIGM-HF (Prospective Comparison of ARNI with ACE inhibitor to Determine Impact on Global Mortality and Morbidity in Heart Failure) study, sacubitril-valsartan led to significantly lower all-cause and cardiovascular mortality of heart failure with reduced ejection fraction (HFrEF) compared to enalapril alone (6). Subsequent researches on ARNI also confirmed this (7–9). In addition, sacubitril-valsartan plays an important role in anti-hypertension (10–14) and renal protection, regardless of baseline renal function (15). Therefore, ARNI is recommended by the International Society of Hypertension Global Hypertension Practice Guidelines for patients with HF or hypertension (16). However, the effectiveness and safety of ARNI have not been well evaluated in patients with severe renal insufficiency (those with glomerular filtration rate <30 ml/min/1.73 m^2^).

Recently, sacubitril-valsartan was applied in advanced chronic kidney disease (CKD) patients (stage IV or V) with HFrEF in a real-world clinical setting, and the positive results were supported by lower overall mortality, cardiovascular death, and re-hospitalization (9). In addition, Tang et al. (8) had undertaken a cohort study focused on peritoneal dialysis (PD) patients with HFpEF (HF with preserved ejection fraction). Of the 21 patients analyzed at last, HF was greatly improved after sacubitril-valsartan administration, not only in clinical signs and symptoms, but also in biochemical indicators. However, data on ARNI treatment in PD patients are still lacking, and the mechanisms of ARNI on HF remain unclear.

Following oral administration, sacubitril-valsartan is rapidly hydrolyzed *in vivo* by carboxyl esterase 1 to the active NEPI, sacubitril, which could inhibit NEP, enhance natriuretic peptide (NP) system activity, and suppress RAAS activation (2–5). Besides cardiovascular (reducing systemic vascular resistance and ventricular preload) and neuro-endocrine (inhibiting sympathetic nerve input and increasing vagus nerve input) activities, sacubitril-valsartan could enhance diuretic and natriuretic actions, regulate sodium-water balance, and improve blood volume (17–19). However, this might not work in end-stage renal disease (ESRD) patients due to severe renal injury and anuria. In PD patients, a major way to remove fluid out of the body is peritoneal ultrafiltration (PUF), especially in those without residual renal function (RRF) (20). Failure of PUF is closely related to water retention and overload, hypertension resistance (21), pulmonary edema (22), and acute or congestive heart failure (CHF) (23). Could ARNI affect PUF? To validate it, we conducted this short-term study involving 47 PD patients.

## Methods

### Study Design and Patients

This was a short-term retrospective self-controlled study enrolled ESRD patients who underwent PD more than 3 month, were over 18 years, and received sacubitril-valsartan in the Department of Nephrology, Nanfang Hospital, Southern Medical University from June 1st 2020 to June 30th 2021. All patients were treated with continuous ambulatory PD (CAPD) or automated PD (APD). Patients with acute infection, trauma, autoimmune disease, active rheumatic diseases, or complicated with severe HF {New York Heart Association (NYHA) functional class III or IV, or serum NT-proBNP ≥11,215.5 pg/ml (24)}, or those transferred to hemodialysis or kidney transplantation within 1 month were excluded. Sacubitril-valsartan was administered after consultation with nephrologist. ACE inhibitors and ARBs were required to be discontinued 36 h before prescribing sacubitril-valsartan. The dose of sacubitril-valsartan was usually 100 mg b.i.d. (25) and no patients discontinued the drug during follow-up. Treatments including dialysis modality, frequency, dialysate glucose concentration, or anti-hypertensive drugs other than ACE inhibitors/ARB did not change. Other drugs were also continued. The study protocol was approved by the research ethics committee of Nanfang Hospital, Southern Medical University (Ethics number NFEC-2019-223), and all participants provided written informed consent.

### Data Collection

Baseline demographic and clinical parameters prior to sacubitril-valsartan administration, including age, gender, body mass index (BMI), PD vintage, dialysate glucose concentration, weekly Kt/V (weekly urea clearance index), primary kidney disease, medical histories, laboratory data and drug use were obtained from medical records and inspection systems. Daily clinical parameters including PUF, urine volume (UV), total output, body weight and blood pressure (BP) were collected within 7 days before and after sacubitril-valsartan treatment.

Conventional weekly Kt/V was measured by standard methods (26). Dialysate glucose concentration (%) was calculated as Σ (glucose concentration×input volume)/total input volume. For example, if a patient is treated by CAPD with 1.5% dialysate×2*L* × 2 and 2.5% dialysate×2*L* × 2, the dialysate glucose concentration equals to 2.0% [(1.5% ×2*L* ×2 + 2.5% × 2*L* × 2)/8*L*]. Total output was calculated as PUF plus UV. Changes of PUF (ΔUltrafiltration) = [ΣPUF after ARNI application (PUF after) – ΣPUF before ARNI application (PUF before)]/7. Changes of UV (ΔUV), total output (ΔTotal output), body weight (Δbody weight), Systolic BP (ΔSBP), and diastolic BP (ΔDBP) were calculated as the same method.

### Statistical Analysis

All statistical analyses were performed using Statistical Package for the Social Sciences (SPSS) version 22.0 for Windows and RStudio software, version 4.0.2. Descriptive results of continuous variables were presented as mean ± SD or medians and interquartile ranges (IQRs), and categorical variables were reported as percentages and numbers. Paired sample *t*-test (normal distribution data) and Wilcoxon paired signed rank test (non-parametric data) were used to compare the self-matching data of PUF, UV, total output, body weight, and BP. The differences of PUF, UV, total output, body weight, and BP before and after ARNI treatment were shown as pseudo-median or mean and 95% confidence interval (CI). The pseudo-median is calculated through Wilcoxon signed rank test with continuity correction (27, 28). All tests were two-tailed, and a *P* < 0.05 was considered statistically significant.

## Results

### Baseline Characteristics

From June 2020 to June 2021, 50 PD patients were recruited. Among them, one patient developed a floating tube, one was complicated with severe HF, one was dropped due to personal reason, and 47 patients have entered the last analysis (Supplementary Figure 1). As shown in Table 1, the mean age was 45.9 ± 12.4-year-old, male/female proportion was 28/19, median BMI was 21.6 (20.4, 23.1) kg/m^2^, and median PD vintage was 27.0 (6.0, 51.0) months. The mean left ventricular ejection fraction (LVEF) was 66.5 ± 9.0%. The underlying kidney diseases were chronic glomerulonephritis (55.3%), diabetic kidney disease (6.4%), hypertensive nephropathy (19.1%), obstructive nephropathy (4.3%), and others (14.9%).

**Table 1:** Baseline characteristics of PD patients before ARNI application.

**Variables**	**All patients (*N* = 47)**
**Demographics**	
Age, year	45.9 ± 12.4
Gender, male/female	28/19
BMI, kg/m^2^	21.6 (20.4, 23.1)
**PD characteristics**	
CAPD, *n* (%)	41 (87.2)
Dialysate GLUC, %	1.9 (1.5, 2.0)
PD vintage, months	27.0 (6.0, 51.0)
Weekly Kt/V	2.0 (1.8, 2.5)
**Causes of ESRD**	
Chronic glomerulonephritis, *n* (%)	26 (55.3)
Diabetic kidney disease, *n* (%)	3 (6.4)
Hypertensive nephropathy, *n* (%)	9 (19.1)
Obstructive nephropathy, *n* (%)	2 (4.3)
Others	7 (14.9)
**Medical history**	
Hypertension, *n* (%)	42 (89.4)
Diabetes mellitus, *n* (%)	10 (21.3)
**Laboratory values**	
Blood hemoglobin, g/L	100.4 ± 16.6
Serum albumin, g/L	36.8 (33.2, 40.9)
Serum creatinine, μmoL/L	918.0 (731.0, 1,178.0)
NT-proBNP, pg/ml	3,732.5 (940.1, 9,563.8)
LVEF, %	66.5 ± 9.0
**Medication use**	
ACE inhibitors or ARBs, *n* (%)	27 (57.4)
Diuretics, *n* (%)	12 (25.5)

*Values are mean ± SD, proportion or interquartile range (IQR). ARNI, angiotensin receptor-neprilysin inhibitor; BMI, body mass index; PD, peritoneal dialysis; CAPD, continuous ambulatory PD; GLUC, glucose concentration; Weekly Kt/V, weekly fractional clearance index for urea; ESRD, end-stage renal disease; NT-proBNP, N-terminal-proB-type natriuretic peptide; LVEF, left ventricular ejection fraction; ACE inhibitors, angiotensin-converting enzyme inhibitors; ARBs, angiotensin II receptor blockers*.

### Changes of PUF, Total Output, UV and Body Weight After Sacubitril-Valsartan Initiating

**Figure 1:**
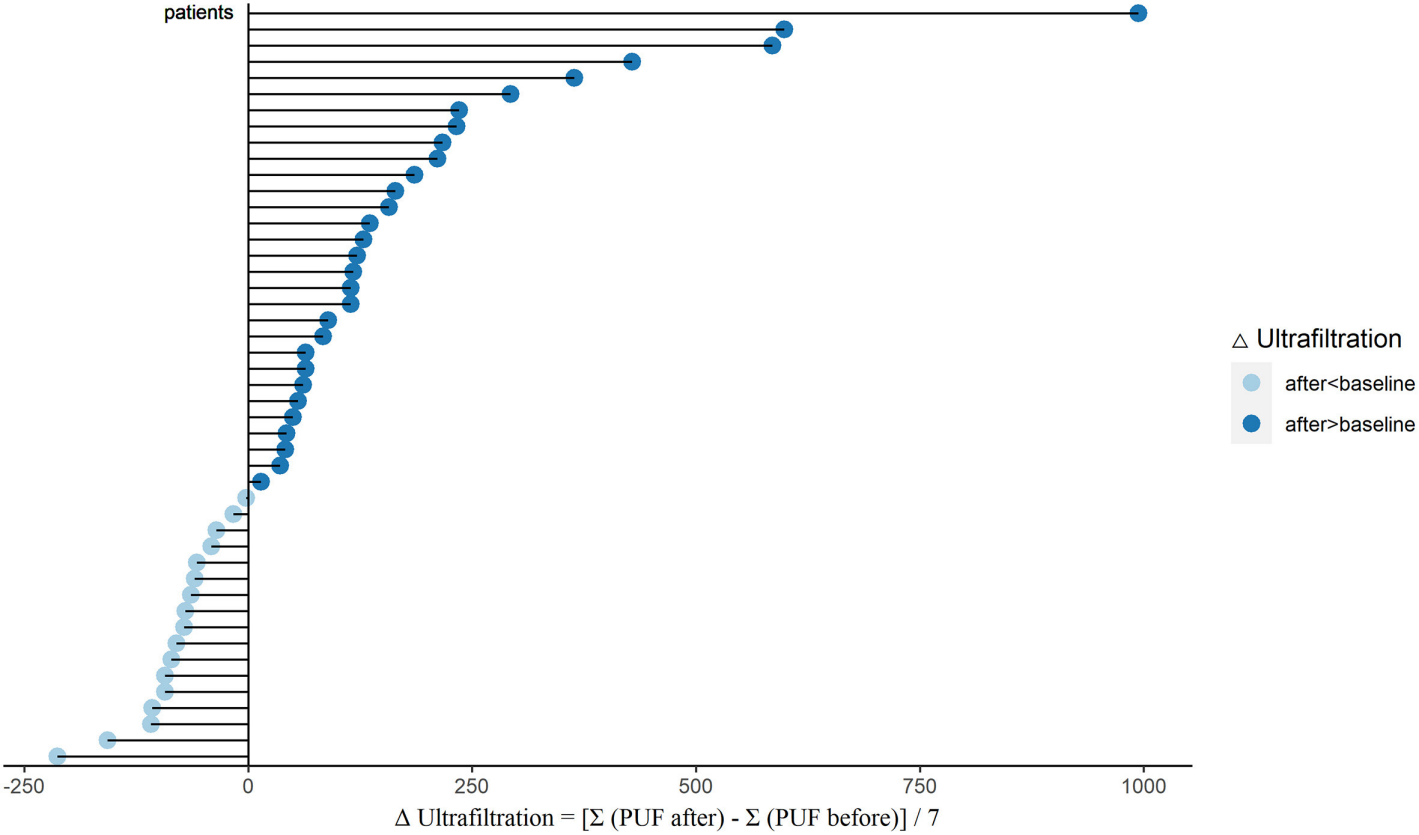
Changes of PUF (ΔUltrafiltration) after sacubitril-valsartan initiating in PD patients. Daily PUF was collected within 7 days before (PUF before) and after (PUF after) sacubitril-valsartan treatment. ΔUltrafiltration = [Σ(PUF after) –Σ (PUF before)]/7. After treated with sacubitril-valsartan, 30 patients had obvious increase of PUF (dark blue color), and 17 patients had slight decrease (light blue color). PUF, peritoneal ultrafiltration; PD, peritoneal dialysis.

**Figure 2:**
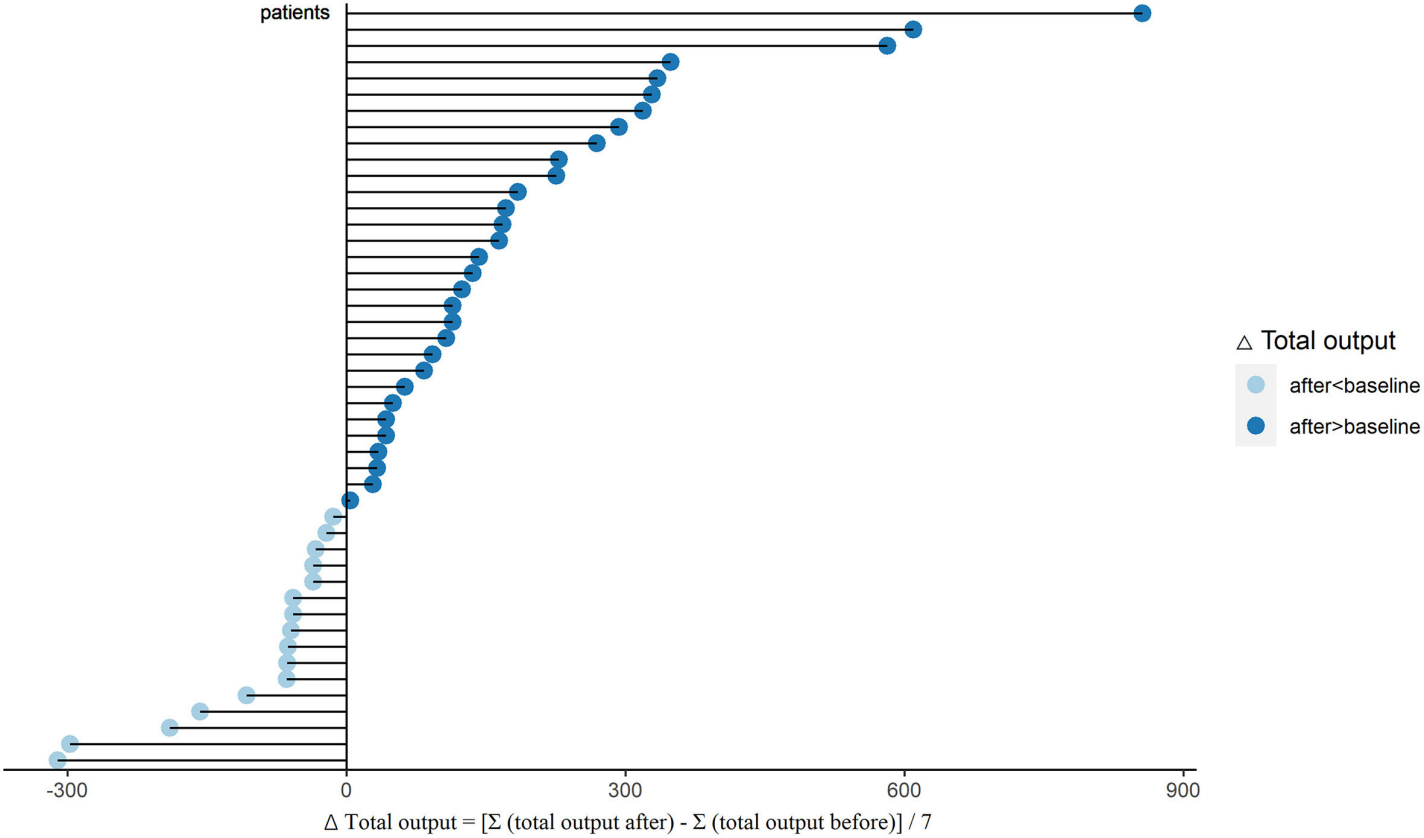
Changes of total output (ΔTotal output) after sacubitril-valsartan initiating in PD patients. Daily total output was collected within 7 days before (total output before) and after (total output after) sacubitril-valsartan treatment. ΔTotal output = [Σ(total output after) – Σ(total output before)]/7. After treated with sacubitril-valsartan, 31 patients had obvious increase of total output (dark blue color), and 16 patients had slight decrease (light blue color). PD, peritoneal dialysis.

To evaluate the water clearance status, we analyzed the changes of PUF, UV, and total output in these PD patients. After sacubitril-valsartan treatment, 30 (63.8%) patients had a significant increase in PUF, while the left 17 (36.2%) decreased slightly (Figure 1; Supplementary Table 1). The pseudo-median daily PUF increase was 66.4 (21.4, 123.2) ml in all PD patients, and 150.4 (110.7, 232.1) ml in the 30 patients (Table 2; Supplementary Table 1). For the total output (PUF plus UV), 31 patients had increased volume with the pseudo-median of 171.4 (114.3, 232.1) ml/24 h, the left 16 patients had decreased slightly [−66.9 (−165.0, −46.3) ml/24 h], and the overall increase was 81.1 (28.6, 139.3) ml/24 h (Table 2, Figure 2; Supplementary Table 1). No significant differences existed in UV (Table 2; Supplementary Figure 2). Therefore, the body weight also decreased [−0.4 (−0.7, −0.1) kg/d, *P* = 0.005, Table 2; Supplementary Figure 5]. Changes of daily PUF, UV, and total output for every patient were shown in Supplementary Figure 3. These data suggested that sacubitril-valsartan could increase PUF and total output in PD patients.

**Table 2:** Comparison of the PUF, Total output, UV, Body weight and BP in PD patients before and after ARNI initiating.

**Variables**	**Before ARNI**	**After ARNI**	**Difference**	***P*-value**
Abnormal distribution*				
PUF, ml/24 h	389.1 (255.4, 536.6)	485.7 (318.6, 647.5)	66.4 (21.4, 123.2)	0.004
Total output, ml/24 h	836.3 (739.3, 919.3)	905.7 (807.9, 1,002.9)	81.1 (28.6, 139.3)	0.003
UV, ml/24 h	532.1 (418.6, 657.1)	520.0 (378.6, 670.7)	20.0 (−41.4, 61.4)	0.446
**Normal distribution** ^‡^				
Body weight, kg	60.0 (56.3, 63.8)	59.6 (56.0, 63.2)	−0.4 (−0.7, −0.1)	0.005
SBP, mmHg	144.6 (139.0, 150.1)	138.7 (133.5, 143.5)	−5.9 (−8.8, −3.0)	<0.001
DBP, mmHg	91.9 (88.4, 95.5)	89.5 (85.9, 93.0)	−2.4 (−4.6, −0.3)	0.030

* *Abnormal distribution, using Wilcoxon paired signed rank test, a non-parametric 95%CI and an estimator for the pseudo-median of the difference of the location parameters is computed. The calculation of the p-value was based on the range of pseudo-median of the distribution of difference. Accordingly, the data before and after ARNI were expressed as pseudo-median (95% CI).*

‡ *Normal distribution, Using paired sample t-test, a 95%CI and an estimator for the mean of the difference is computed. The calculation of the p-value was based on the range of the mean of difference. Accordingly, the data before and after ARNI were expressed as mean (95% CI).*

*PUF, peritoneal ultrafiltration; UV, urine volume; BP, blood pressure; PD, peritoneal dialysis; ARNI, angiotensin receptor-neprilysin inhibitor; SBP, systolic blood pressure; DBP, diastolic blood pressure; CI, confidence interval*.

### Changes of BP Under Sacubitril-Valsartan Treatment

**Figure 3:**
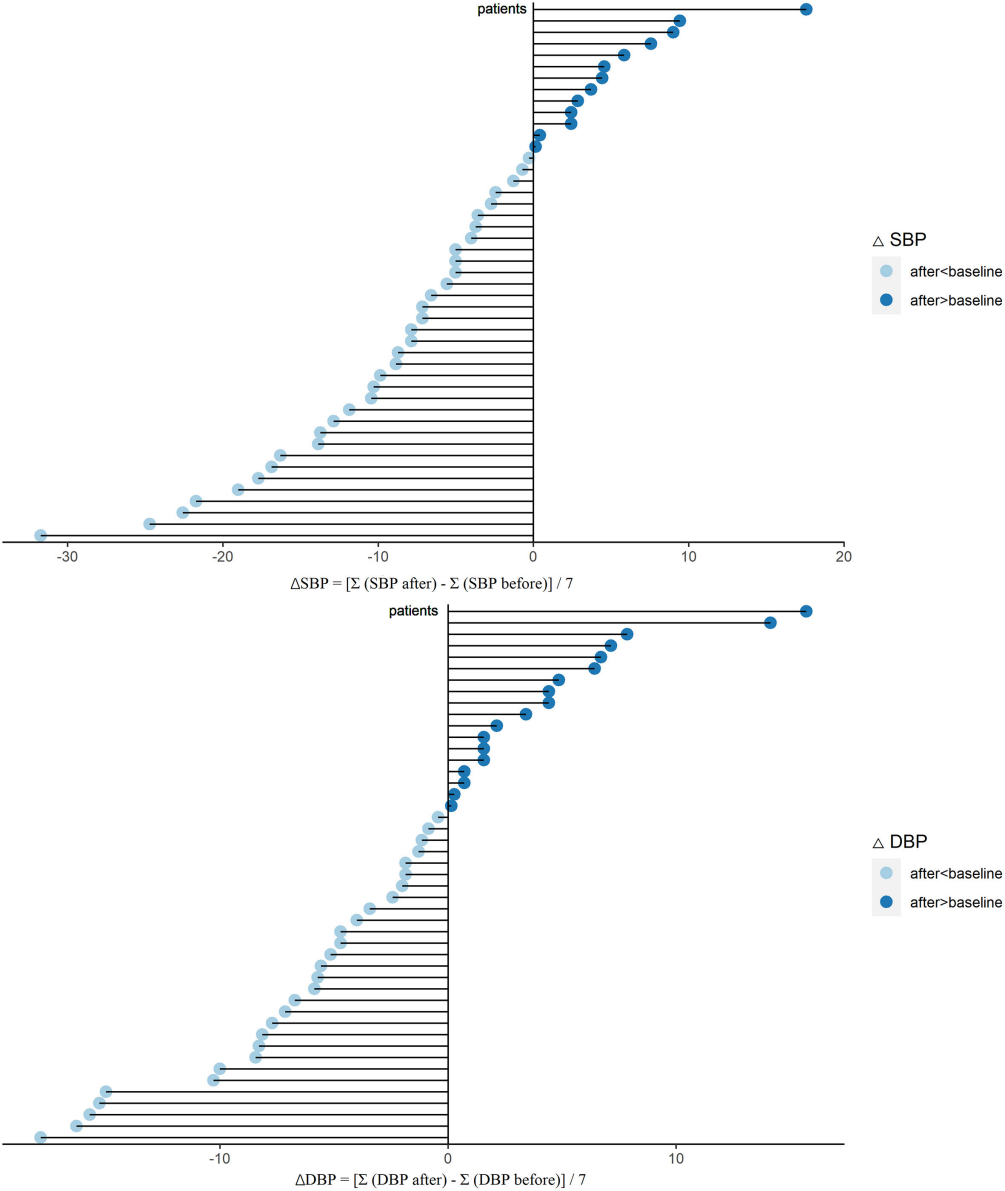
Changes of SBP (ΔSBP) and DBP (ΔDBP) after sacubitril-valsartan initiating in PD patients. Daily BP level was collected during 7 days before (SBP or DBP before) and after (SBP or DBP after) sacubitril-valsartan treatment. ΔSBP = [Σ(SBP after) – Σ(SBP before)]/7. ΔDBP = [Σ(DBP after) – Σ(DBP before)]/7. After treated with sacubitril-valsartan, the BP levels of PD patients were markedly decreased (light blue color). SBP, systolic blood pressure; DBP, diastolic blood pressure; PD, peritoneal dialysis.

Hypertension management is another outstanding role of ARNI (10). Then we established the effect of sacubitril-valsartan in BP. After sacubitril-valsartan treatment, the SBP level did decrease with the mean of −5.9 (−8.8, −3.0) mmHg (*P* < 0.001), and the DBP level also decreased (Table 2, Figure 3). Before receiving sacubitril-valsartan, one patient received ACE inhibitor, 26 patients received ARB, and no patient was treated with ACE inhibitor and ARB combination. Among them, the SBP levels also significantly improved (*P* = 0.017) (Supplementary Table 1). Detailed BPs within 7 days before and after sacubitril-valsartan treatment were shown in Supplementary Figure 4.

### Safety of Sacubitril-Valsartan

We also established the safety of sacubitril-valsartan in this short-term study. None of the PD patients showed adverse drug reactions such as hypotension, hyperkalaemia, or angioedema (Table 3). These data were similar to those reported by Tang et al. (8), but seemed to be lower than those reported in HF population (Table 3) (6).

**Table 3:** Adverse events during sacubitril-valsartan treatment.

**Events**	McMurray et al.*(*N* = 4,203) (%)	Tang et al.**(*N* = 21) (%)	**Current study (*N* = 47) (%)**
Hypotension	17.6	0	0
Hyperkalaemia	11.6	0	0
Cough	8.8	0	0
Renal impairment	10.1	0	0
Angioedema	0.5	0	0

* *McMurray et al. (6).*

** *Fu et al. (8)*.

## Discussion

In this short-term self-controlled study, we have evaluated the role of sacubitril-valsartan on PUF in PD patients. After receiving sacubitril-valsartan, 30 patients had a great increase in PUF, reaching 150.4 ml per day. The total output also increased. Both systolic and diastolic BP significantly decreased. These data demonstrated the role of sacubitril-valsartan in water removal and BP control in PD patients.

HF is a major global public health problem that affects more than 64 million people worldwide (29). Substantial data demonstrated that HF is more common in CKD patients compared to those without CKD (30–33), and the prevalence increases with the renal function progression (31, 33, 34). In ESRD patients undergoing dialysis, the HF incidence increases to 12–36 times of the general population (31, 34). Even though PD is a kind of continuous dialysis model, patients are prone to suffer from CHF due to water-sodium retention (35), which are mainly caused by inefficient water removal (36), especially in those with peritonitis and micro-inflammation (37), long term high glucose exposure (38), peritoneal injury, and peritoneal fibrosis (39, 40). Full water removal and proper blood volume control are very important to improve cardiac function in this population (35). The most important way for water removal in PD patients is PUF because of RRF loss. Interestingly, the PUF of our 47 PD patients did increase rapidly after sacubitril-valsartan application. Within the 7 days after ARNI treatment, about 2/3 patients had a significant PUF increase with a pseudo-median daily volume of 150.4 ml. Of the left decreased 1/3 patients, the PUF changed slightly (−75.0 ml/24 h). The UV did not change after sacubitril-valsartan prescription, which might be caused by poor RRF (baseline pseudo-median UV was 532.1 ml, and 12 patients were anuria). Anyway, the total output was increased. These data suggested that sacubitril-valsartan could improve water removal through increasing PUF, but not urine. Extra fluid removal of ARNI might contribute to cardiac function protection in PD population.

At present, more studies have proved the role of sacubitril-valsartan on cardiac function (6–9), which might be the potential mechanism for better PUF. However, obvious improvement of cardiac function could be generally detected 1–3 months or even longer after ARNI application (41, 42). To ensure the safety, patients with severe HF at baseline have been excluded in our study, including those with NYHA functional class III or IV, or those with serum NT-proBNP ≥ 11,215.5 pg/ml [due to the prolonged half-life and increased plasma concentration of NT-proBNP with the kidney function injury progression (43), and comprehensive consideration of sensitivity and specificity in ESRD patients (24)]. Their mean baseline LVEF score was 66.5 ± 9.0 %. Most importantly, the changes of PUF were observed in a very short term (within 7 days) after sacubitril-valsartan administration. Taken together, although not retested, the effect of sacubitril-valsartan on PUF in PD patients seemed to be associated with ARNI itself, but not with the improvement of cardiac function. How ARNI affects PUF?

Sacubitril-valsartan could rapidly hydrolyze to sacubitril and valsartan after oral intake. Sacubitril could inhibit NEP, enhance NP system activity, and then exerting many biological activities (2–5), including extra water removal though renal tubular NEPI-NP activity. NEP, the zinc-dependent enzyme and type II integral membrane protein, could widely express on many kinds of epi- and endothelial cells (renal, lung, heart, blood vessels, and so on) (44). Peritoneum, a key functional structure for PD, is mainly composed by capillary endothelial cells and peritoneal mesothelial cells (a special epithelial cell). Even though there were no evidence of NEP on peritoneal expression, inhibiting peritoneal endo-/epithelial cellular, NEP might be a potential mechanism for sacubitril-valsartan on PUF. Further cellular and animal experiments are needed for this hypothesis.

The excellent anti-hypertensive effect of ARNI has been confirmed recently (9–14). Compared to ACE inhibitor or ARB alone, ARNI acts synergistically with RAAS blockade, which leads additional anti-hypertensive activation (4, 5). In our study, 27 PD patients were converted to sacubitril-valsartan from ACE inhibitor / ARB, and the other 20 patients were added directly. All of them achieved better BP levels. Extra water removal by higher ultrafiltration and special activity of ARNI might be the reasons. However, the amount of anti-hypertensive drugs after sacubitril-valsartan treatment was higher than the original scheme (generally 2 tablets vs. 1 tablet in patients switched from ACE inhibitor/ARB, or 2 tablets vs. 0 tablet in patients added directly), which might be another key factor. Further comparative trails of ARNI vs. ACE inhibitor/ARB in a same dose for BP control are needed.

In existing studies, the common adverse drug reactions of ARNI are symptomatic hypotension, cough, renal impairment, hyperkalemia, and angio-oedema (6). In our study, no PD patients have shown any adverse drug reaction. However, this might be mainly caused by very short-term observation (only 7 days after applying sacubitril-valsartan) and relatively small sample size (47 participants). The real side effects of sacubitril-valsartan in PD patients should be fully evaluated in the subsequent longer-term and larger sample size studies.

To our knowledge, data on ARNI treatment in PD patients are limited. Here we found that sacubitril-valsartan might improve PUF and BP in PD patients, and the self-controlled study setting could remove some confounding factors. However, there were several limitations. The first limitation is the sample size. Even though the sample size is larger than reported before (47 vs. 21 patients), it is still relatively small, which might bring us some errors. The second limitation is the observation period. We have only observed 7 days after ARNI administration. This short-term observation gives us some detailed dynamic information and some hints, but could not predict long-term effects. We were unable to evaluate the effect of ARNI on cardiac function in this short observation period. Furthermore, side effects of ARNI in PD population could not be fully established. Third, prescription of ARNI was 200 mg per day according to the HF guidelines (25) and was higher than the original scheme, which might be a key factor of better BP control. Finally, retrospective cohort study did bring us some inevitable confounding factors. It is necessary to conduct larger sample size, longer term, prospective cohort, and randomized double-blinded controlled studies to confirm the effect of ARNI on PUF and cardiac function in PD patients.

## Conclusions

Our study suggested that sacubitril-valsartan was associated with the increase of short-term PUF and total output in PD patients. This is a first study about the relationship between ARNI and extra water removal in PD patients. If further confirmed, ARNI application might bring us a potential method to improve water retention and cardiac function in PD population.

## Data Availability Statement

The raw data supporting the conclusions of this article will be made available by the authors, without undue reservation.

## Ethics Statement

The studies involving human participants were reviewed and approved by Ethics Committee of Nanfang Hospital (Ethics Number NFEC-2019-223). The patients/participants provided their written informed consent to participate in this study.

## Author Contributions

JA, FZ, and TZ made substantial contributions to the conception and design of the study. FZ, TZ, SY, DW, and QZ were responsible for the acquisition of data, analysis, and interpretation of data. FZ and JA were involved in drafting the manuscript. JA, NG, and XQ were responsible for revising the manuscript critically for important intellectual content. All authors contributed to the article and approved the submitted version.

## Funding

This work was supported by the Nature and Science Foundation of China (No. 81770765) and Outstanding Youths Development Scheme of Nanfang Hospital, Southern Medical University (No. 2017J013) to JA.

## Conflict of Interest

The authors declare that the research was conducted in the absence of any commercial or financial relationships that could be construed as a potential conflict of interest.

## Publisher's Note

All claims expressed in this article are solely those of the authors and do not necessarily represent those of their affiliated organizations, or those of the publisher, the editors and the reviewers. Any product that may be evaluated in this article, or claim that may be made by its manufacturer, is not guaranteed or endorsed by the publisher.

## References

[1] Ksander GM Ghai RD DeJesus R Diefenbacher CG Yuan A Berry C. Dicarboxylic acid dipeptide neutral endopeptidase inhibitors. J Med Chem. (1995) 38:1689–700. 10.1021/jm00010a014 7752193

[2] Gu J Noe A Chandra P Al-Fayoumi S Ligueros-Saylan M Sarangapani R. Pharmacokinetics and pharmacodynamics of LCZ696, a novel dual-acting angiotensin receptor-neprilysin inhibitor (ARNi). J Clin Pharmacol. (2010) 50:401–14. 10.1177/0091270009343932 19934029

[3] Shi J Wang X Nguyen J Wu AH Bleske BE Zhu HJ. Sacubitril is selectively activated by carboxylesterase 1 (CES1) in the liver and the activation is affected by CES1 genetic variation. Drug Metab Dispos. (2016) 44:554–9. 10.1124/dmd.115.068536 26817948 PMC4810765

[4] Solomon SD Zile M Pieske B Voors A Shah A Kraigher-Krainer E. The angiotensin receptor neprilysin inhibitor LCZ696 in heart failure with preserved ejection fraction: a phase 2 double-blind randomised controlled trial. Lancet. (2012) 380:1387–95. 10.1016/S0140-6736(12)61227-6 22932717

[5] Iborra-Egea O Gálvez-Montón C Roura S Perea-Gil I Prat-Vidal C Soler-Botija C. Mechanisms of action of sacubitril/valsartan on cardiac remodeling: a systems biology approach. NPJ Syst Biol Appl. (2017) 3:12. 10.1038/s41540-017-0013-4 28649439 PMC5460292

[6] McMurray JJV Packer M Desai AS Gong J Lefkowitz MP Rizkala AR. Angiotensin–neprilysin inhibition versus enalapril in heart failure. N Engl J Med. (2014) 371:993–1004. 10.1056/NEJMoa1409077 25176015

[7] Haynes R Judge PK Staplin N Herrington WG Storey BC Bethel A. Effects of sacubitril/valsartan versus irbesartan in patients with chronic kidney disease. Circulation. (2018) 138:1505–14. 10.1161/CIRCULATIONAHA.118.034818 30002098

[8] Fu S Xu Z Lin B Chen J Huang Q Xu Y. Effects of sacubitril-valsartan in heart failure with preserved ejection fraction in patients undergoing peritoneal dialysis. Front Med. (2021) 8:657067. 10.3389/fmed.2021.657067 34235161 PMC8255468

[9] Chang HY Feng AN Fong MC Hsueh CW Lai WT Huang KC. Sacubitril/valsartan in heart failure with reduced ejection fraction patients: real world experience on advanced chronic kidney disease, hypotension, and dose escalation. J Cardiol. (2019) 74:372–80. 10.1016/j.jjcc.2019.03.010 30982680

[10] Ruilope LM Dukat A Bohm M Lacourciere Y Gong J Lefkowitz MP. Blood-pressure reduction with LCZ696, a novel dual-acting inhibitor of the angiotensin II receptor and neprilysin: a randomised, double-blind, placebo-controlled, active comparator study. Lancet. (2010) 375:1255–66. 10.1016/S0140-6736(09)61966-8 20236700

[11] Kario K Sun N Chiang FT Supasyndh O Baek SH Inubushi-Molessa A. Efficacy and safety of LCZ696, a first-in-class angiotensin receptor neprilysin inhibitor, in Asian patients with hypertension: a randomized, double-blind, placebo-controlled study. Hypertension. (2014) 63:698–705. 10.1161/HYPERTENSIONAHA.113.02002 24446062

[12] Ito S Satoh M Tamaki Y Gotou H Charney A Okino N. Safety and efficacy of LCZ696, a first-in-class angiotensin receptor neprilysin inhibitor, in Japanese patients with hypertension and renal dysfunction. Hypertens Res. (2015) 38:269–75. 10.1038/hr.2015.1 25693859 PMC4396400

[13] Kario K Tamaki Y Okino N Gotou H Zhu M Zhang J. LCZ696, a First-in-class angiotensin receptor-neprilysin inhibitor: the first clinical experience in patients with severe hypertension. J Clin Hypertens. (2016) 18:308–14. 10.1111/jch.12667 26402918 PMC8032009

[14] Huo Y Li W Webb R Zhao L Wang Q Guo W. Efficacy and safety of sacubitril/valsartan compared with olmesartan in Asian patients with essential hypertension: a randomized, double-blind, 8-week study. J Clin Hypertens. (2018) 20:150–8. 10.1111/jch.13437 30536595 PMC8030324

[15] Damman K Gori M Claggett B Jhund PS Senni M Lefkowitz MP. Renal effects and associated outcomes during angiotensin-neprilysin inhibition in heart failure. JACC Heart Fail. (2018) 6:489–98. 10.1016/j.jchf.2018.02.004 29655829

[16] Unger T Borghi C Charchar F Khan NA Poulter NR Prabhakaran D. 2020 International society of hypertension global hypertension practice guidelines. Hypertension. (2020) 75:1334–57. 10.1161/HYPERTENSIONAHA.120.15026 32370572

[17] Vardeny O Miller R Solomon SD. Combined neprilysin and renin-angiotensin system inhibition for the treatment of heart failure. JACC Heart Fail. (2014) 2:663–70. 10.1016/j.jchf.2014.09.001 25306450

[18] Bayes-Genis A Morant-Talamante N Lupón J. Neprilysin and natriuretic peptide regulation in heart failure. Curr Heart Fail Rep. (2016) 13:151–7. 10.1007/s11897-016-0292-x 27260315

[19] Wang T Tan R Lee H Ihm S Rhee M Tomlinson B. Effects of sacubitril/valsartan (LCZ696) on natriuresis, diuresis, blood pressures, and NT-proBNP in salt-sensitive hypertension. Hypertension. (2017) 69:32–41. 10.1161/HYPERTENSIONAHA.116.08484 27849566

[20] Krediet RT Douma CE van Olden RW. Ho-dac-Pannekeet MM, Struijk DG. augmenting solute clearance in peritoneal dialysis. Kidney Int. (1998) 54:2218–25. 10.1046/j.1523-1755.1998.00181.x 9853288

[21] Vaios V Georgianos PI Liakopoulos V Agarwal R. Assessment and management of hypertension among patients on peritoneal dialysis. Clin J Am Soc Nephro. (2019) 14:297–305. 10.2215/CJN.07480618 30341090 PMC6390915

[22] Almeida CP Ponce D de Marchi AC Balbi AL. Effect of peritoneal dialysis on respiratory mechanics in acute kidney injury patients. Perit Dial Int. (2014) 34:544–9. 10.3747/pdi.2013.00092 25074997 PMC4114672

[23] Bertoli SV Musetti C Ciurlino D Basile C Galli E Gambaro G. Peritoneal ultrafiltration in refractory heart failure: a cohort study. Perit Dial Int:. (2014) 34:64–70. 10.3747/pdi.2012.00290 24179103 PMC3923694

[24] Jafri L Kashif W Tai J Siddiqui I Azam I Shahzad H. B-type natriuretic peptide versus amino terminal pro-B type natriuretic peptide: selecting the optimal heart failure marker in patients with impaired kidney function. BMC Nephrol. (2013) 14:117. 10.1186/1471-2369-14-117 23725445 PMC3680180

[25] Seferovic PM Ponikowski P Anker SD Bauersachs J Chioncel O Cleland JGF. Clinical practice update on heart failure 2019: pharmacotherapy, procedures, devices and patient management. an expert consensus meeting report of the heart failure association of the European society of cardiology Eur J Heart Fail. (2019) 21:1169–86. 10.1002/ejhf.1531 31129923

[26] NKF-DOQI clinical practice guidelines for the treatment of anemia of chronic renal failure. national kidney foundation-dialysis outcomes quality initiative. Am J Kidney Dis. (1997) 30:S192–240. 9339151

[27] Vaquero-Martinez J Anton M Ortiz DGJ Cachorro VE Wang H Gonzalez AG. Validation of integrated water vapor from OMI satellite instrument against reference GPS data at the Iberian Peninsula. Sci Total Environ. (2017) 580:857–64. 10.1016/j.scitotenv.2016.12.032 27988187

[28] Hollander M DA Wolfe. Nonparametric Statistical Methods. New York, NY: Wiley (1973). p. 34.

[29] Groenewegen A Rutten FH Mosterd A Hoes AW. Epidemiology of heart failure. Eur J Heart Fail. (2020) 22:1342–56. 10.1002/ejhf.1858 32483830 PMC7540043

[30] Tuegel C Bansal N. Heart failure in patients with kidney disease. Heart. (2017) 103:1848–53. 10.1136/heartjnl-2016-310794 28716974

[31] Kottgen A Russell SD Loehr LR Crainiceanu CM Rosamond WD Chang PP. Reduced kidney function as a risk factor for incident heart failure: the atherosclerosis risk in communities (ARIC) study. J Am Soc Nephrol. (2007) 18:1307–15. 10.1681/ASN.2006101159 17344421

[32] Bansal N Katz R Robinson-Cohen C Odden MC Dalrymple L Shlipak MG. Absolute rates of heart failure, coronary heart disease, and stroke in chronic kidney disease: an analysis of 3 community-based cohort studies. JAMA Cardiol. (2017) 2:314. 10.1001/jamacardio.2016.4652 28002548 PMC5832350

[33] Mann JF Gerstein HC Pogue J Bosch J Yusuf S. Renal insufficiency as a predictor of cardiovascular outcomes and the impact of ramipril: the hope randomized trial. Ann Intern Med. (2001) 134:629–36. 10.7326/0003-4819-134-8-200104170-00007 11304102

[34] Foley RN. Clinical epidemiology of cardiac disease in dialysis patients: left ventricular hypertrophy, ischemic heart disease, and cardiac failure. Semin Dialysis. (2003) 16:111–7. 10.1046/j.1525-139X.2003.160271.x 12641874

[35] Lu R Muciño-Bermejo M Ribeiro LC Tonini E Estremadoyro C Samoni S. Peritoneal dialysis in patients with refractory congestive heart failure: a systematic review. Cardiorenal Med. (2015) 5:145–56. 10.1159/000380915 25999963 PMC4427136

[36] Fusshoeller A. Histomorphological and functional changes of the peritoneal membrane during long-term peritoneal dialysis. Pediatr Nephrol. (2008) 23:19–25. 10.1007/s00467-007-0541-z 17638023

[37] Noh H Kim JS Han KH Lee GT Song JS Chung SH. Oxidative stress during peritoneal dialysis: implications in functional and structural changes in the membrane. Kidney Int. (2006) 69:2022–8. 10.1038/sj.ki.5001506 16641917

[38] Sitter T Sauter M. Impact of glucose in peritoneal dialysis: saint or sinner? Perit Dial Int. (2005) 25:415–25. 10.1177/089686080502500502 16178471

[39] Williams JD Craig KJ Topley N Von Ruhland C Fallon M Newman GR. Morphologic changes in the peritoneal membrane of patients with renal disease. J Am Soc Nephrol. (2002) 13:470–9. 10.1681/ASN.V132470 11805177

[40] Selgas R Bajo A Jiménez-Heffernan JA Sánchez-Tomero JA Del Peso G Aguilera A. Epithelial-to-mesenchymal transition of the mesothelial cell—its role in the response of the peritoneum to dialysis. Nephrol Dial Transpl. (2006) 21:i2–7. 10.1093/ndt/gfl183 16825254

[41] Velazquez EJ Morrow DA DeVore AD Duffy CI Ambrosy AP McCague K. Angiotensin-neprilysin inhibition in acute decompensated heart failure. N Engl J Med. (2019) 380:539–48. 10.1056/NEJMoa1812851 30415601

[42] Morrow DA Velazquez EJ DeVore AD Desai AS Duffy CI Ambrosy AP. Clinical outcomes in patients with acute decompensated heart failure randomly assigned to sacubitril/Valsartan or enalapril in the PIONEER-HF trial. Circulation. (2019) 139:2285–8. 10.1161/CIRCULATIONAHA.118.039331 30955360

[43] Takase H Dohi Y. Kidney function crucially affects B-type natriuretic peptide (BNP), N-terminal proBNP and their relationship. Eur J Clin Invest. (2014) 44:303–8. 10.1111/eci.12234 24372567

[44] Nalivaeva NN Zhuravin IA Turner AJ. Neprilysin expression and functions in development, ageing and disease. Mech Ageing Dev. (2020) 192:111363. 10.1016/j.mad.2020.111363 32987038 PMC7519013

