# Supplementary material for: Sacubitril-Valsartan Increases Ultrafiltration in Patients Undergoing Peritoneal Dialysis: A Short-Term Retrospective Self-Controlled Study
Source: Front Med (Lausanne). 2022 Jun 3;9:831541. doi: 10.3389/fmed.2022.831541 (PMC9203730; doi:10.3389/fmed.2022.831541)
Supplement: Supplementary file 1 [file Data_Sheet_1.ZIP › Supplementary Figure legends.docx]

**Supplementary Figure legends：**

**Supplementary Figure 1.** **Flow chart of enrollment and exclusion.** PD, peritoneal dialysis; HF, heart failure.

**Supplementary Figure 2. Changes of UV (Δ UV) after sacubitril-valsartan initiating in PD patients.** Daily UV was collected within 7 days before (UV before) and after (UV after) sacubitril-valsartan treatment. Δ UV = [Σ (UV after) - Σ (UV before)] / 7. Of the 47 patients with PD, 12 had anuria. After treated with sacubitril-valsartan, 20 patients had increase of UV (dark blue color), and 15 patients had slight decrease (light blue color). UV, urine volume; PD, peritoneal dialysis.

**Supplementary Figure 3. Longitudinal changes of daily PUF, UV, and total output for every PD patient within 7 days before and after sacubitril-valsartan** **initiating.** The detailed changes of daily PUF, UV, and total output of each patient within 7 days before and after sacubitril-valsartan initiating were presented. PUF, peritoneal ultrafiltration; UV, urine volume; PD, peritoneal dialysis.

**Supplementary Figure 4. Changes of BP for every patient within 7 days before and after sacubitril-valsartan initiating.** The detailed changes of the daily BP of each patient within 7 days before and after sacubitril-valsartan initiating were presented. BP, blood pressure; PD, peritoneal dialysis.

**Supplementary Figure 5. Changes of** **body weight (Δ Body weight) after sacubitril-valsartan initiating in PD patients.** Daily body weight was collected within 7 days before (body weight before) and after (body weight after) sacubitril-valsartan treatment. Δ Body weight = [Σ (body weight after) - Σ (body weight before)] / 7. Of the 47 PD patients, 11 had only baseline body weight. After treated with sacubitril-valsartan, 27 patients had decrease of body weight (light blue color), and 9 patients had slight increase (dark blue color). PD, peritoneal dialysis.
