# Supplementary material for: Sacubitril-Valsartan Increases Ultrafiltration in Patients Undergoing Peritoneal Dialysis: A Short-Term Retrospective Self-Controlled Study
Source: Front Med (Lausanne). 2022 Jun 3;9:831541. doi: 10.3389/fmed.2022.831541 (PMC9203730; doi:10.3389/fmed.2022.831541)
Supplement: Supplementary file 1 [file Data_Sheet_1.ZIP › Supplementary Table 1.docx]

**Supplementary Table 1.** **Comparison of the detailed PUF, Total output and SBP in PD patients before and after ARNI initiating.**

| **Variables** | **Before ARNI** | **After ARNI** | **Difference** | **P-value** |
| --- | --- | --- | --- | --- |
| **PUF, ml/24h***  PUF increases  PUF decreases  **Total output, ml/24h***  total output increases  total output decreases  **SBP, mmHg**‡  received ACEI / ARB  without ACEI / ARB | 394.3 (237.1, 585.7)  379.3 (86.1, 682.9)  813.6 (675.7, 922.1)  889.6 (679.3, 999.3)  149.3 (141.7, 156.9)  138.1 (130.2, 146.1) | 599.3 (403.6, 796.9)  289.3 (-25.7, 608.6)  985.2 (843.6, 1119.4)  780.4 (615.7, 909.3)  143.7 (136.9, 150.4)  131.6 (124.6, 138.5) | 150.4 (110.7, 232.1)  -75.0 (-100.0, -54.6)  171.4 (114.3, 232.1)  -66.9 (-165.0, -46.3)  -5.6 (-10.2, -1.1)  -6.6 (-10.1, -3.0) | <0.001  <0.001  <0.001  <0.001  0.017  0.001 |

Patients with increased PUF were 30, patients with decreased PUF were 17. Patients with increased total output were 31, patients with decreased total output were 16. 27 patients had been previously received ACEI / ARB, 20 patients had not. PUF, peritoneal ultrafiltration; SBP, systolic blood pressure; PD, peritoneal dialysis; ARNI, angiotensin receptor-neprilysin inhibitor; ACEI, angiotensin-converting enzyme inhibitor; ARB, angiotensin II receptor blocker; CI, confidence interval.

* For abnormal distributed data, using Wilcoxon paired signed rank test, a nonparametric 95%CI and an estimator for the pseudo-median of the difference of the location parameters is computed. The calculation of the p-value was based on the range of pseudo-median of the distribution of difference. Accordingly, the data before and after ARNI were expressed as pseudo-median (95% CI).

‡ For normal distributed data, using paired sample t-test, a 95%CI and an estimator for the mean of the difference is computed. The calculation of the p-value was based on the range of the mean of difference. Accordingly, the data before and after ARNI were expressed as mean (95% CI).
