# Supplementary figures and images for: Sacubitril-Valsartan Increases Ultrafiltration in Patients Undergoing Peritoneal Dialysis: A Short-Term Retrospective Self-Controlled Study
Source: Front Med (Lausanne). 2022 Jun 3;9:831541. doi: 10.3389/fmed.2022.831541 (PMC9203730; doi:10.3389/fmed.2022.831541)

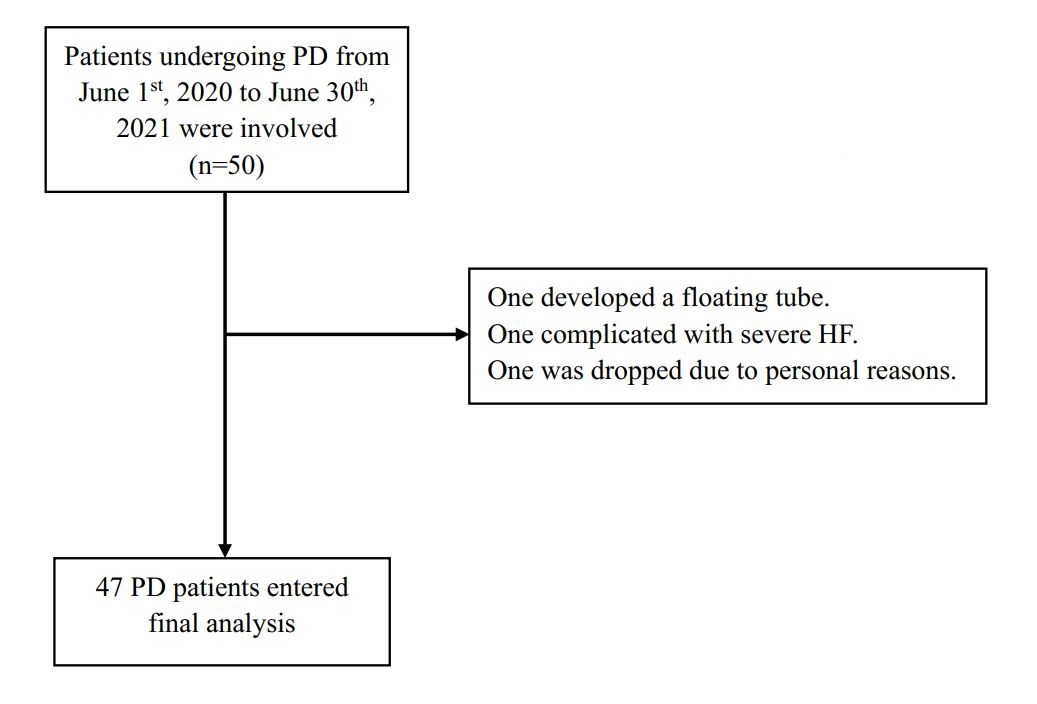

Supplement: Supplementary file 1 [file Data_Sheet_1.ZIP › Supplementary Figure 1.JPG]

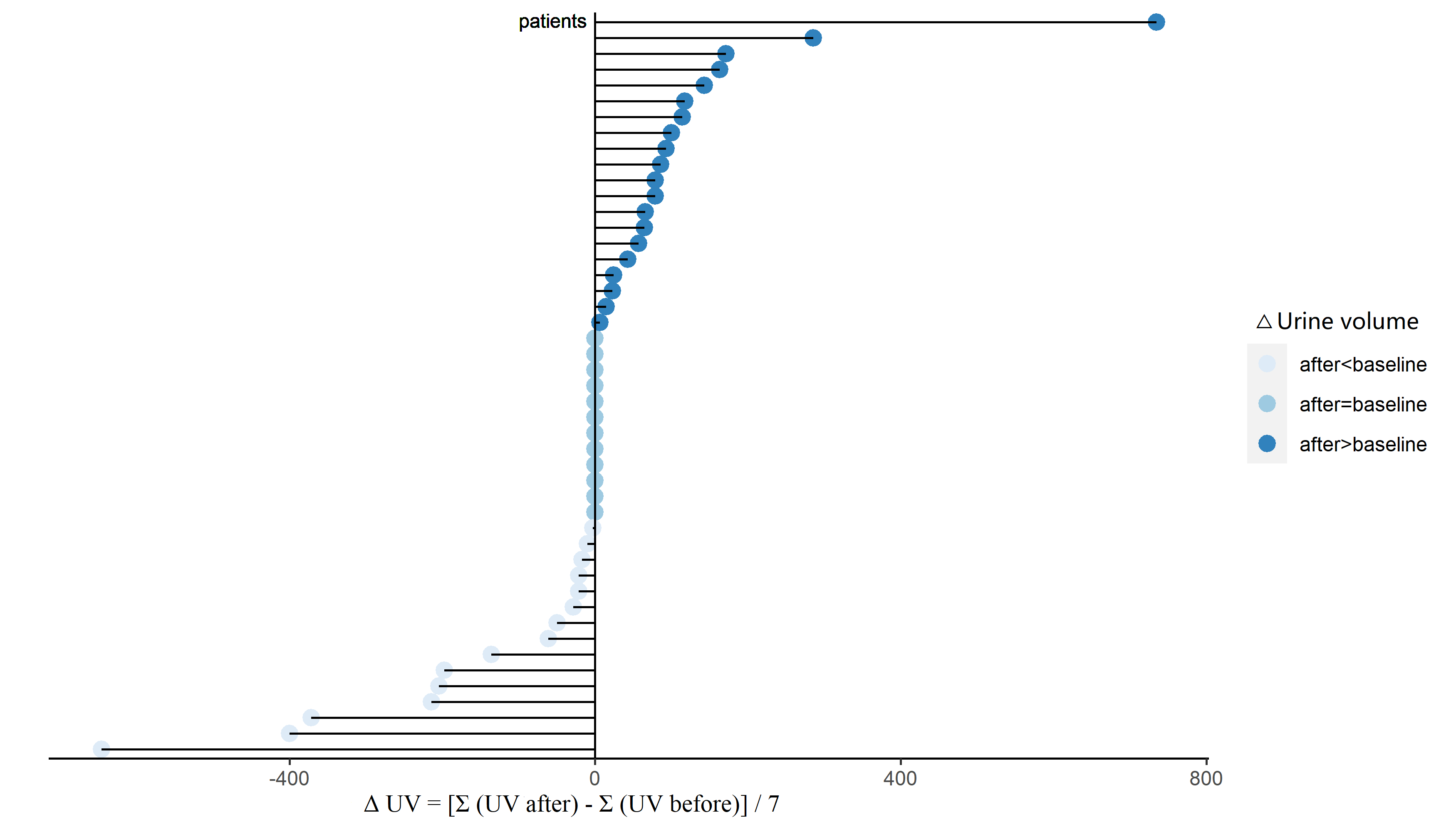

Supplement: Supplementary file 1 [file Data_Sheet_1.ZIP › Supplementary Figure 2.tiff]

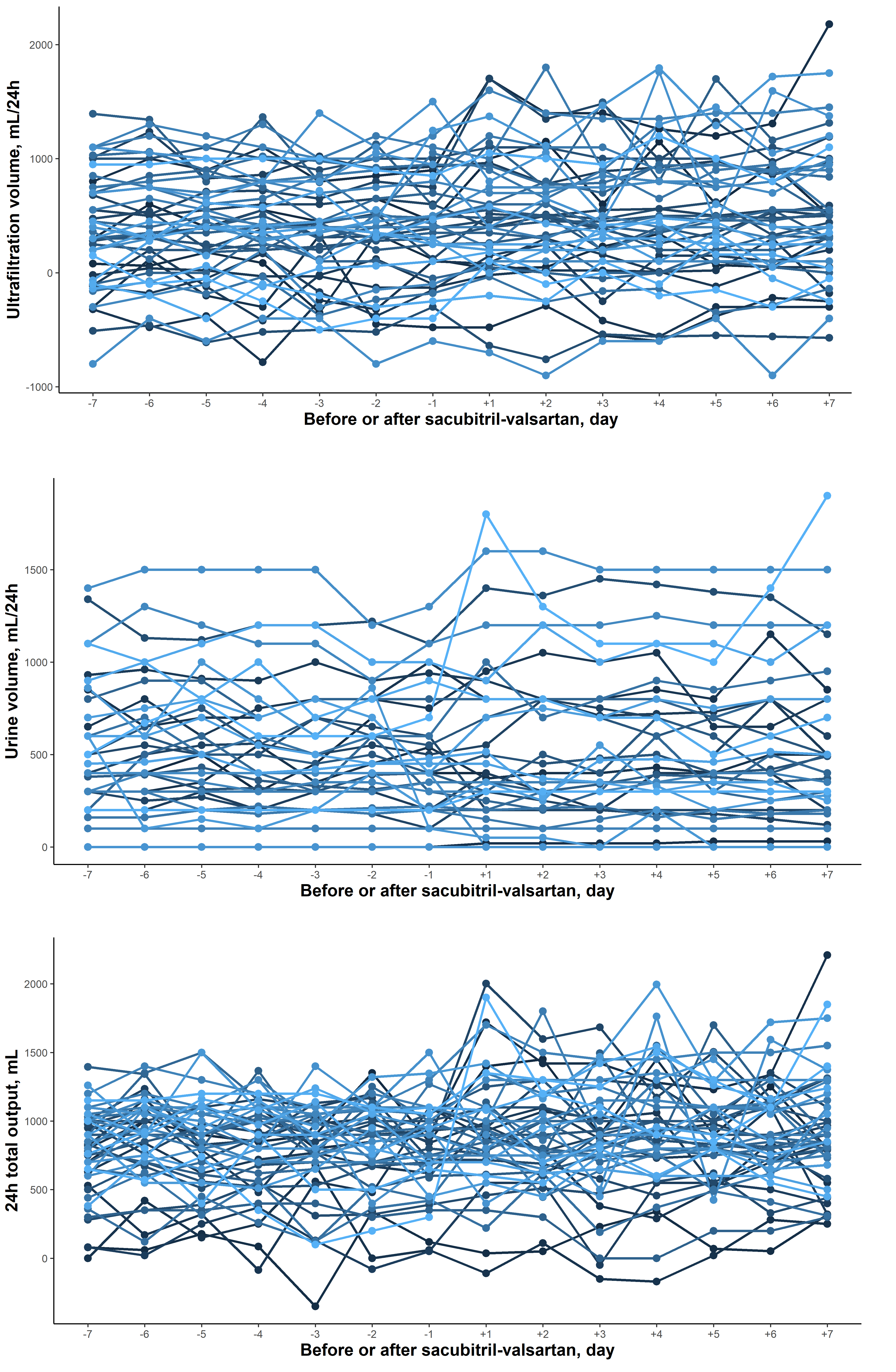

Supplement: Supplementary file 1 [file Data_Sheet_1.ZIP › Supplementary Figure 3.tif]

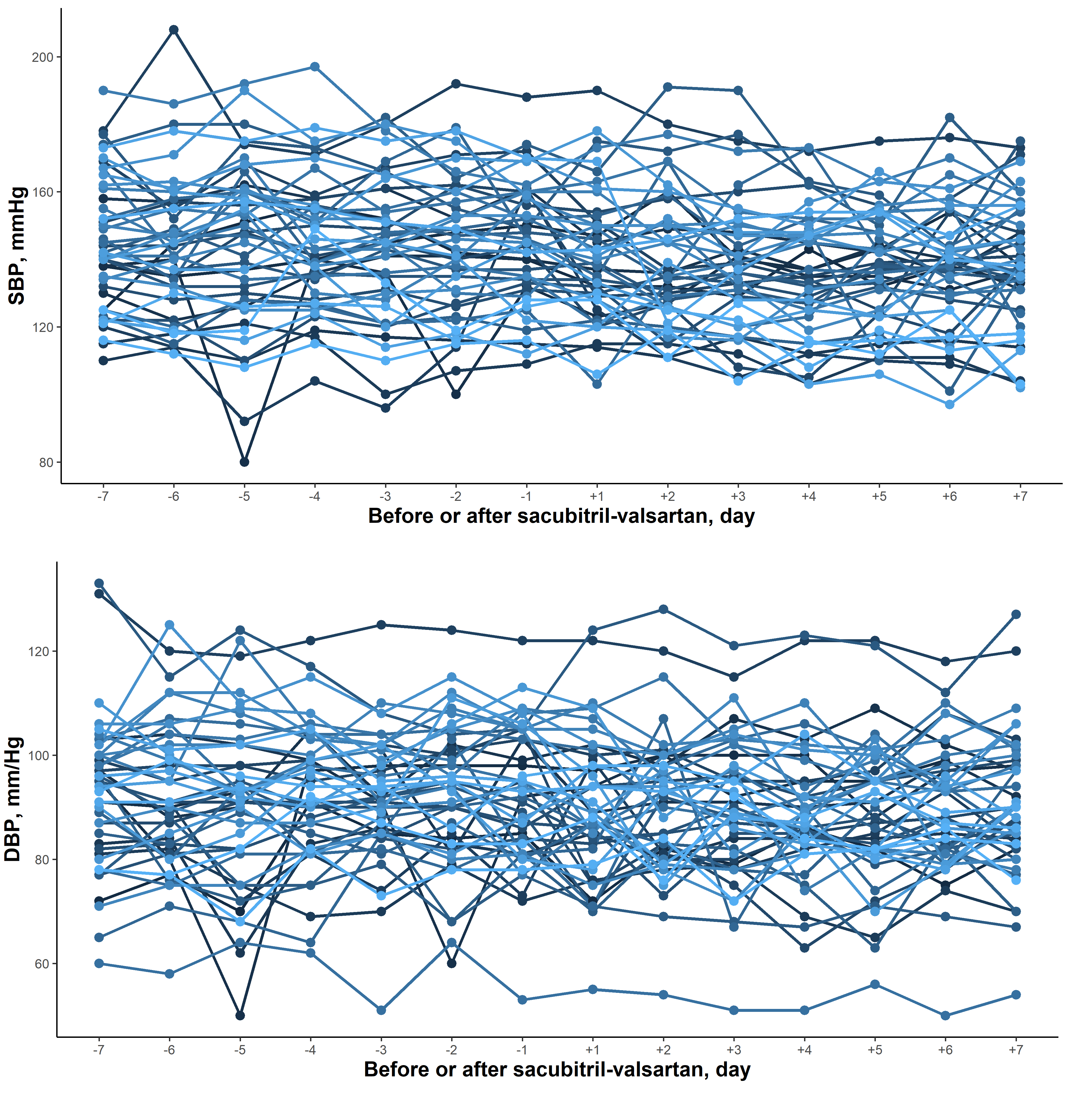

Supplement: Supplementary file 1 [file Data_Sheet_1.ZIP › Supplementary Figure 4.tif]

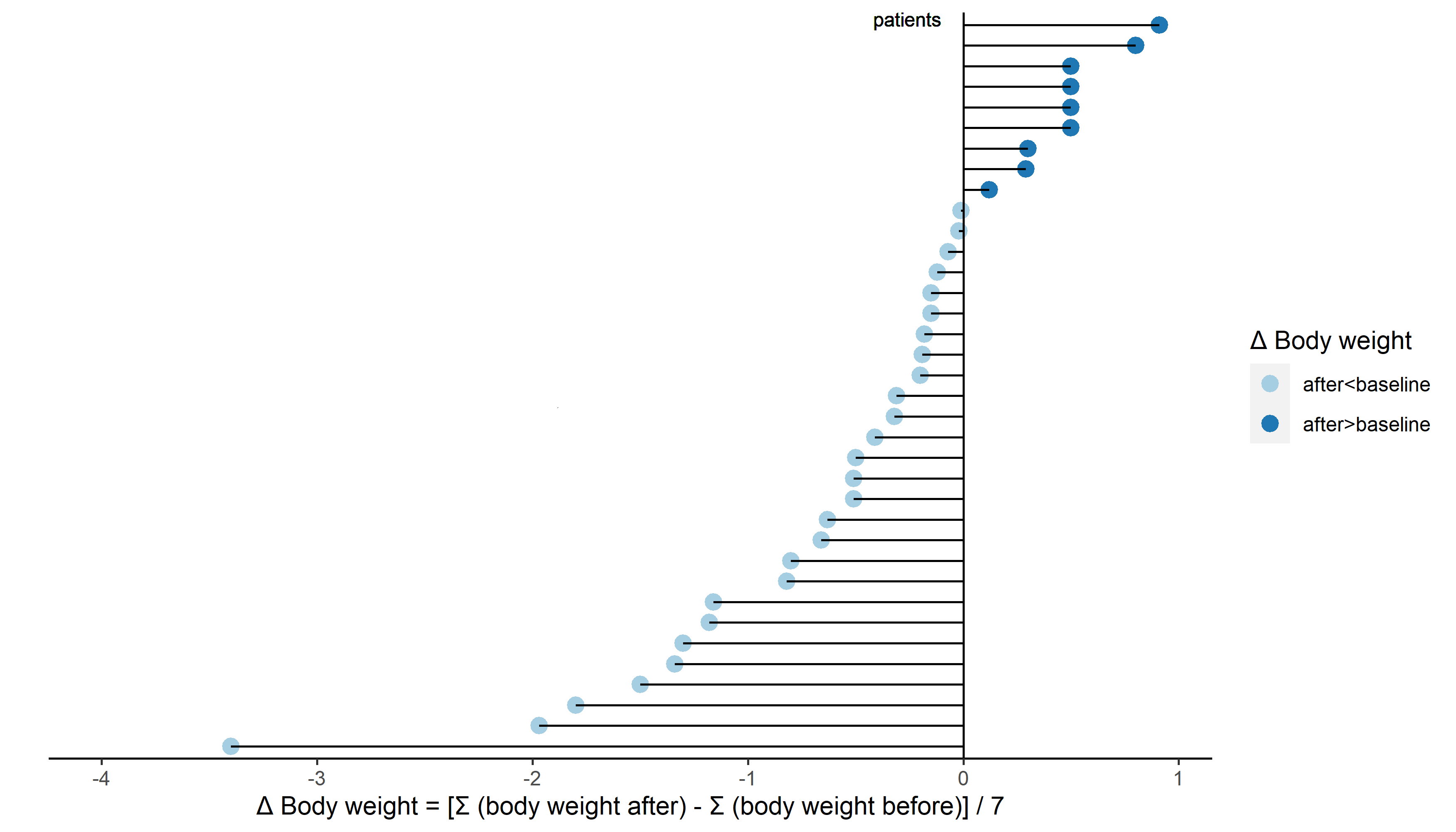

Supplement: Supplementary file 1 [file Data_Sheet_1.ZIP › Supplementary Figure 5.tiff]
